# Severe Orbital Myiasis Caused by *Chrysomya bezziana*: A Case Report

**DOI:** 10.4274/tjo.galenos.2020.00225

**Published:** 2021-02-25

**Authors:** Yu Siang Ng, Yuen Keat Gan, Leni Tupang

**Affiliations:** 1 Ministry of Health Malaysia, Hospital Keningau, Department of Ophthalmology, Sabah, Malaysia; 2 Entomology & Pest Unit, Public Health Keningau, Ministry of Health Malaysia, Sabah, Malaysia

**Keywords:** Chrysomya bezziana, orbital myiasis, maggots, exenteration

## Abstract

An 88-year-old woman was brought to the hospital immediately after her neighbours noticed that she was bleeding from her right eye. On examination, her right eye was phthisic with maggot infestation of her right orbit. Over a hundred live maggots were extracted using forceps. Computed tomography scan revealed the infestation was confined to the right orbit. The patient underwent exenteration of the right orbit under general anaesthesia. The species was identified by an entomologist as *Chrysomya bezziana*, which has aggressive larvae that eat living tissue. This case report demonstrates that orbital myiasis caused by *C. bezziana* poses a very real risk of intracranial invasion as they feed on living tissues. Adjacent tissue destruction can be very rapid and definitive treatment involves urgent removal of its larvae via surgical debridement. To our knowledge, we are reporting the first case of orbital myiasis from a patient in Malaysia. Therefore, our case report may be helpful in the management of similar case of orbital myiasis.

## Introduction

Myiasis, derived from the Greek word *mya* which means fly, is a condition caused by infestation of dipterous larvae in humans or vertebrate animals. It is a rare condition, mainly seen among elderly, infirm, and immobile patients that are poorly cared for.^1^ Eggs are deposited on an exposed wound or in this case on the eyelid by an adult fly. The larvae can feed on necrotic or living tissues, leading to tissue destruction of the orbit. Identification of the species responsible for a case of myiasis is not commonly done.^2^ We report a case of orbital myiasis in a geriatric patient who was immobile and emaciated in the interior parts of Sabah, Malaysia.

## Case Report

**Figure 1:**
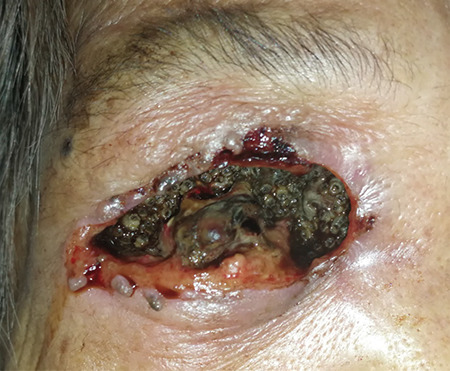
An 88-year-old woman from the interior division of Sabah, Malaysia, presenting with massive maggot infestation of the right orbit

**Figure 2:**
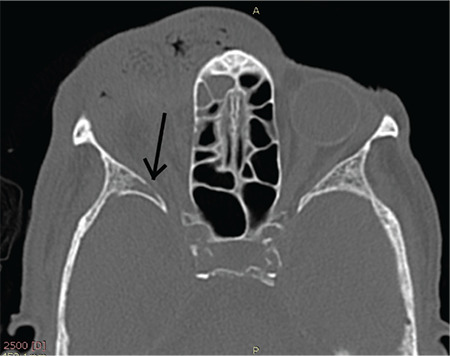
Computed tomography image showing bony erosion of the greater wing of sphenoid of the right orbit caused by *Chrysomya bezziana* larvae

**Figure 3:**
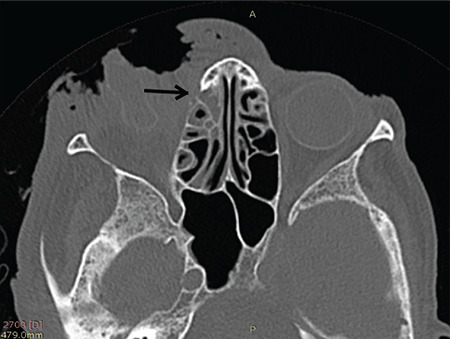
Computed tomography image showing bony erosion of the lamina papyracea of the right orbit caused by *Chrysomya bezziana* larvae

An 88-year-old indigenous Murut woman who was bed-bound with underlying chronic lung disease resided in rural Sabah. She lived with another equally infirm relative with poor social support due to their place of residence in the thick jungle. She had presented to the Hospital Keningau Eye clinic in 2016 with eyelash loss and a dark pigmented growth with central ulceration over the right upper eyelid that bled on contact. Malignancy of the right eyelid was suspected at the time but she refused excisional biopsy of the lesion and was lost to follow-up. Apart from that, she had no known comorbidity such as diabetes mellitus or immunosuppression. Three years later, she was brought to the emergency department by her neighbors due to bleeding from her right eye. On examination, there were numerous live maggots in her right orbit with the orbital bone exposed (Figure 1). The right eye was phthisic and surrounded with necrotic tissue. The fellow eye was erythematous around the eyelids with no maggots seen. She was treated for right orbital myiasis with left preseptal cellulitis. She also presented with acute kidney injury that eventually improved with treatment. Urgent computed tomography showed the myiasis was confined to the right orbit, with no brain or paranasal sinuses involved (Figures 2, 3). Nasoendoscopy and otoscopy showed no myiasis in the sinuses and auditory canal. Intravenous ceftriaxone 1 g once a day and intravenous metronidazole 500 mg 3 times a day were administered for 2 weeks. Daily extraction of live maggots from the wound using forceps was carried out in the ward. More than a hundred maggots were removed bedside prior to surgery. Patient eventually underwent an exenteration of the right orbit under general anesthesia. Intraoperatively there was bony erosion at the right lamina papyracea and greater wing of the right sphenoid bone, but no tumorous tissue or regional lymphadenopathy were apparent. Live maggots were removed intraoperatively on sight. Postoperatively, the right orbital wound was clean with no remaining maggots detected. Unfortunately, patient succumbed to pneumonia 2 weeks after surgery.

**Figure 4:**
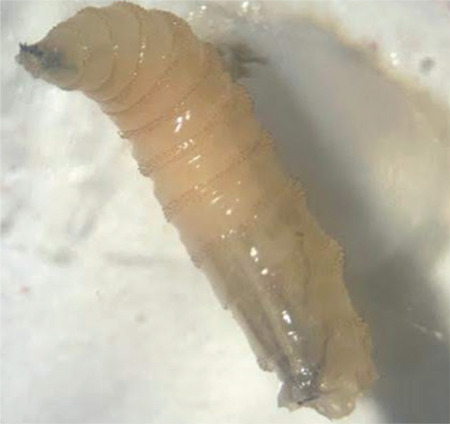
Third instar of *Chrysomya bezziana* larva removed from the patient’s right orbit wound

**Figure 5:**
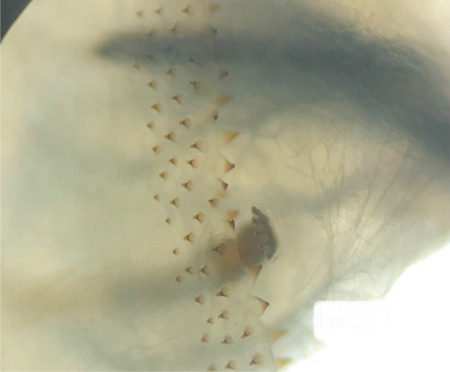
Anterior spiracle (5 fingers) and body spine on the thoracic segments of a *Chrysomya bezziana* larva (x40 magnification)

**Figure 6:**
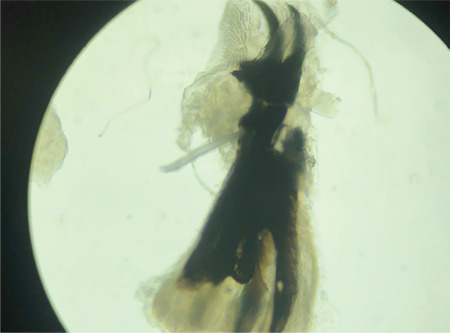
Cephaloskeleton of a *Chrysomya bezziana* larva (x40 magnification)

**Figure 7:**
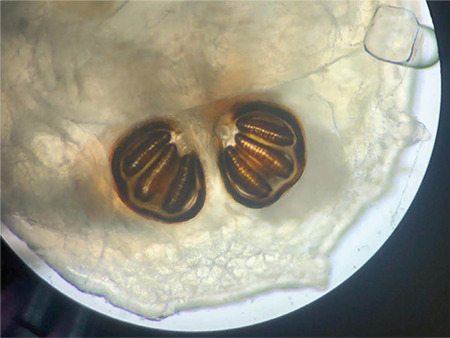
Peritreme of the posterior spiracle of a *Chrysomya bezziana* larva (x40 magnification)

The maggots were identified by an entomologist as *Chrysomya bezziana*. At the entomology & pest unit, macroscopic examination of the larvae revealed worm-like bodies of fully-grown third instars, each measuring approximately 13 mm in length (Figure 4). They had smooth, broad bodies, with cuticular spines along the body segment (Figure 5). Examination was focused on the characteristics of the cephaloskeleton (Figure 6), anterior spiracle with 5 papillae (Figure 5), and a posterior spiracle (Figure 7). The cuticular spines between the first and second thoracic segments were thorn-like black spines with single teeth. All these characteristics were unique to *C. bezziana*, the Old World screwworm fly.^3^

## Discussion

Orbital myiasis is a rare form of eye disease which has been reported in both developed as well as developing countries. A review of the literature revealed that human myiasis is most commonly caused by *Oestrus ovis* (32 cases reported worldwide), followed by *C. bezziana* (15 cases), *Cochliomyia hominivorax* (14 cases), and *Wohlfartia magnifica* (12 cases).^4^

Adult female flies usually lay eggs directly onto necrotic, hemorrhagic, or suppurative tissue. The larvae emerge and feed on organic matter, which can lead to complications such as cornea ulcer, orbital cellulitis, globe invasion, endophthalmitis, blindness, disfigurement, and even death.^1,2^

The adult *C. bezziana* fly is green or blue-green in color and feeds on decaying matter, excreta, and flowers. Approximately 150-200 eggs at a time are laid by the female adult on exposed wounds and mucous membranes of the mouth, ears, and nose. After 24 hours, the eggs hatch and the larvae burrow deep into living tissue in a screw-like fashion, completing their development while feeding on host tissue for 5 to 7 days. Thereafter they fall to the ground to pupate. The pupal stage is temperature-dependent, with sexual maturation in approximately 1 week to 2 months. Thus it takes 2 to 3 months to complete their life cycle.^5^ In the present case, the larvae were creamy white in color, 12-18 mm long, with a worm-like appearance that gradually tapered at the anterior end. The anterior spiracles were palmate in shape, each being composed of 4 to 6 lobes arranged in a single row located at dorso-posterior margin on each side of prothorax. The posterior spiracles consisted of 3 oblique slits encircled by a dark, thick peritreme that is incomplete ventro-medially around the compressed button. With the help of available keys in the literature, the maggots were identified as third instar larvae of *C. bezziana* on the basis of anterior and posterior spiracles and the cephalophyaryngeal apparatus.^5,6^

*C. bezziana* is an obligate parasite of mammals, commonly found in Asia, tropical Africa, India, and Papua New Guinea. In contrast to many other fly larvae that feed on necrotic tissue, *C. bezziana* larvae feed on living tissue of warm-blooded mammals, and hence is capable of deep tissue penetration and rapid destruction. It buries deep in the tissue and remains firmly attached using its cuticular spines. Upon contact with forceps during removal, the larvae tend to escape by burrowing deeper into the tissue, making removal challenging.

In orbital myiasis, the extent of infestation and tissue destruction should be delineated to exclude intracranial spread using computed tomography or magnetic resonance imaging. If orbital involvement is localized and less extensive, non-invasive mechanical removal of the maggots can be done.^3^ Non-tooth forceps are reported to be suitable for larvae removal to avoid breaking the larvae, which may lead to foreign body or allergic reactions.^7^ We found epilation forceps especially useful due to their broad tips for a firmer grasp. We also recommend making circular motions during removal of deeply burrowed larvae, as it eases removal and prevents them from breaking midway. All larvae were removed in toto using this method.

It has also been reported that materials such as petroleum jelly or pork fat helps in asphyxiating larvae, forcing them to protrude to the surface intermittently.^8^ This makes the extraction easier.

The degree of orbital involvement was extensive in our patient, and an exenteration and debridement of necrotic tissue was done to prevent intracranial extension.^1^

There are anecdotal reports that oral or topical ivermectin is beneficial in the treatment of severe orbital myiasis by *C. hominivorax* because it paralyzes the larvae.^9^ However, overall these are insufficient evidence to support the use of ivermectin in *C. bezziana* orbital myiasis.^3^

In conclusion, infestation by this fly species causes rapid tissue destruction and requires urgent definitive treatment via surgical debridement.
